# Supplementary material for: Engineering of ultraID, a compact and hyperactive enzyme for proximity-dependent biotinylation in living cells
Source: Commun Biol. 2022 Jul 4;5:657. doi: 10.1038/s42003-022-03604-5 (PMC9253107; doi:10.1038/s42003-022-03604-5)
Supplement: Supplementary file 2 — Supplementary Information [file 42003_2022_3604_MOESM2_ESM.pdf]

Supplementary Information

Engineering of ultraID, a compact and hyperactive enzyme for proximity-dependent biotinylation in living cells

Lea Kubitz[1,*], Sebastian Bitsch[2,*], Xiyan Zhao[1,*], Kerstin Schmitt[3], Lukas Deweid[2,§], Amélie Roehrig[1,ƒ], Elisa Cappio Barazzone[1ʃ], Oliver Valerius[3], Harald Kolmar[2], Julien Béthune[4,#]
** These authors contributed equally*
# Corresponding author: Julien.bethune@haw-hamburg.de

1: Heidelberg University Biochemistry Center, Heidelberg, Germany
2: Institute for Organic Chemistry and Biochemistry, Technische Universität Darmstadt, Darmstadt, Germany
3: Institute of Microbiology and Genetics, Göttingen Center for Molecular Biosciences (GZMB) and Service Unit LCMS Protein Analytics, Georg-August-University Göttingen, Göttingen, Germany
4: Department of Biotechnology, Hamburg University of Applied Sciences, Hamburg, Germany

[§]Lukas Deweid present address: Ferring Pharmaceuticals, Copenhagen, Denmark
[ƒ]Amélie Roehrig present address: Inserm UMRS1138 – FunGeST team, Paris, France
[ʃ]Elisa Cappio Barazzone present address: ETH Zürich, Department of Health Sciences and Technology, Zürich, Switzerland

This PDF file includes:

Supplementary Figures 1 to 16

Supplementary Tables 1 to 4

Supplementary Note 1

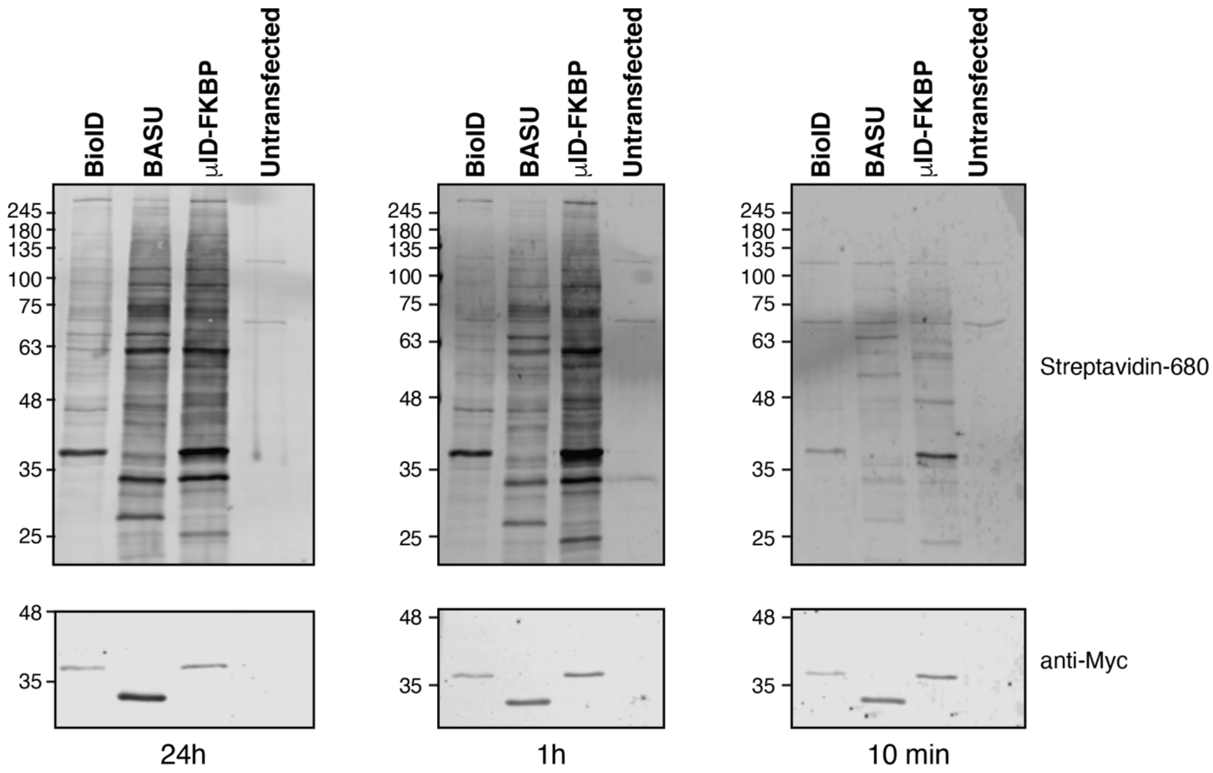

Supplementary Figure 1: microID is an enhanced activity PDB enzyme.
Blots of lysates of HeLa cells transiently transfected with the indicated constructs and incubated with 50 μM biotin for 24 h (left), 1 h (middle) or 10 min (right). Biotinylation was analyzed using IRDye680-labeled streptavidin and expression levels of the fusion proteins with antibodies against the Myc tag as indicated.

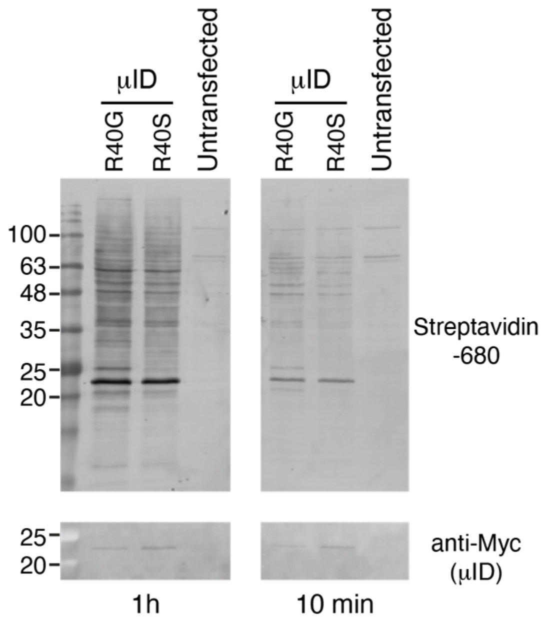

Supplementary Figure 2: The R40S mutation does not enhance the activity of microID.

Blots of lysates of HeLa cells transiently expressing Myc-tagged microID with either the R40G or the R40S mutations and incubated with with 50 μM biotin for 1 h (left) or 10 min (right). Biotinylation was analyzed using IRDye680-labeled streptavidin and expression levels of the fusion proteins with antibodies against the Myc tag as indicated.

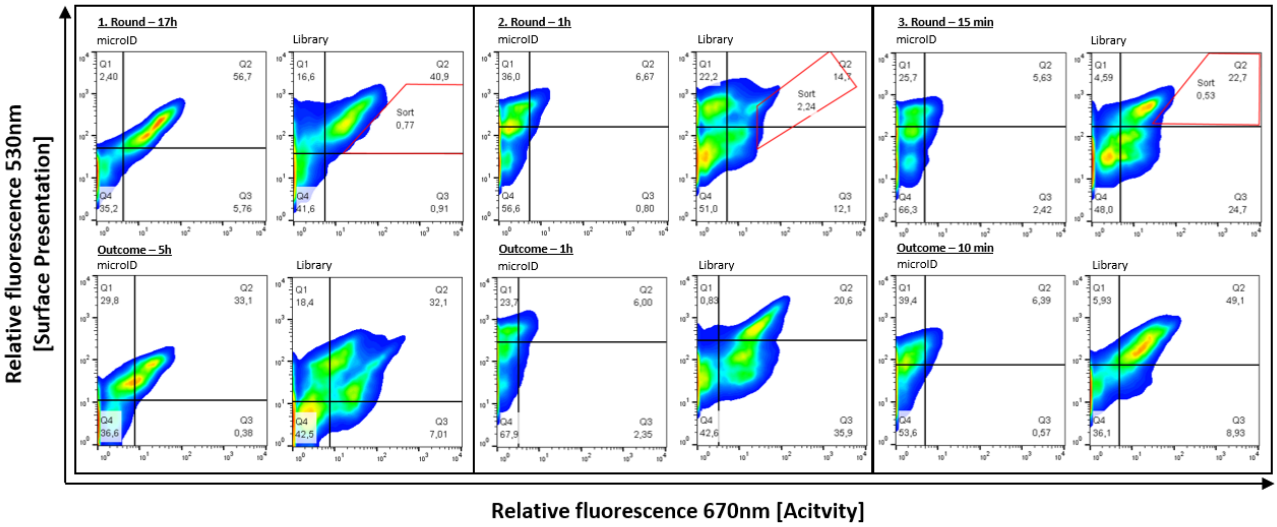

Supplementary Figure 3: Progress of the directed evolution of microID.
Upper row: sorting rounds with indication of the sorting cells (red gates), bottom row: outcome of the cell enrichment after sorting.

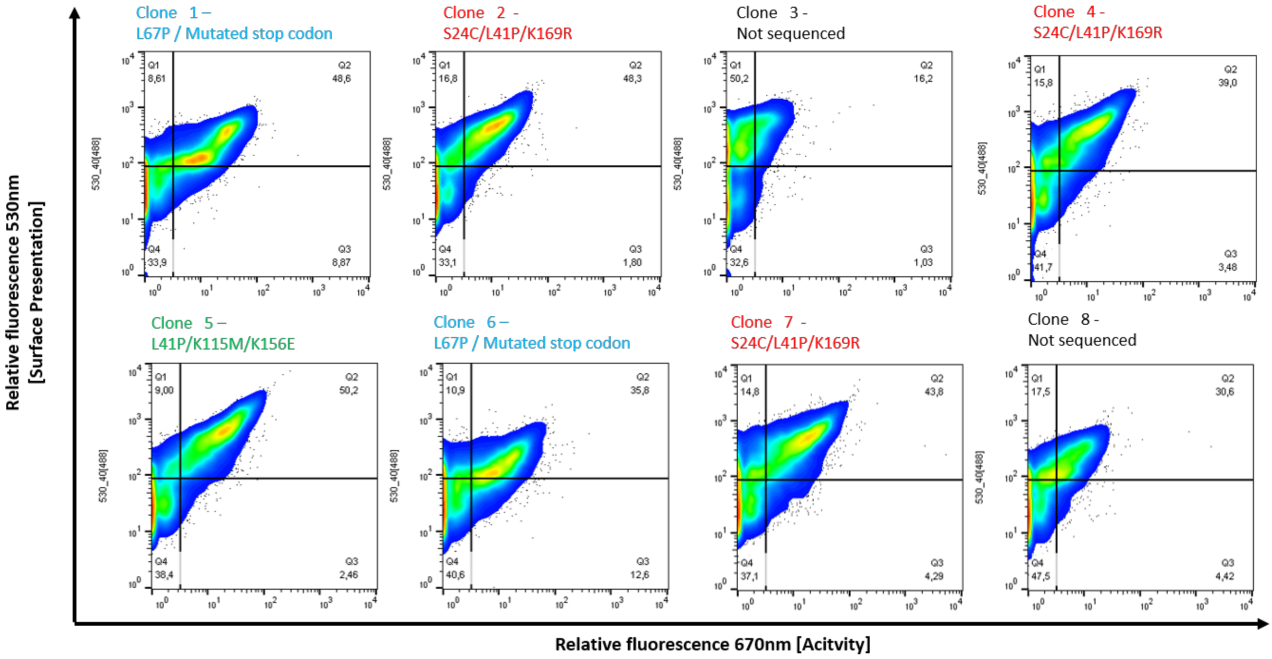

Supplementary Figure 4: Single clone analysis from the round 3 yeast library. The indicated clones were analyzed for surface presentation (y-axis) and biotinylation activity (x-axis). The mutations, deduced by DNA sequencing, on each clone are indicated.

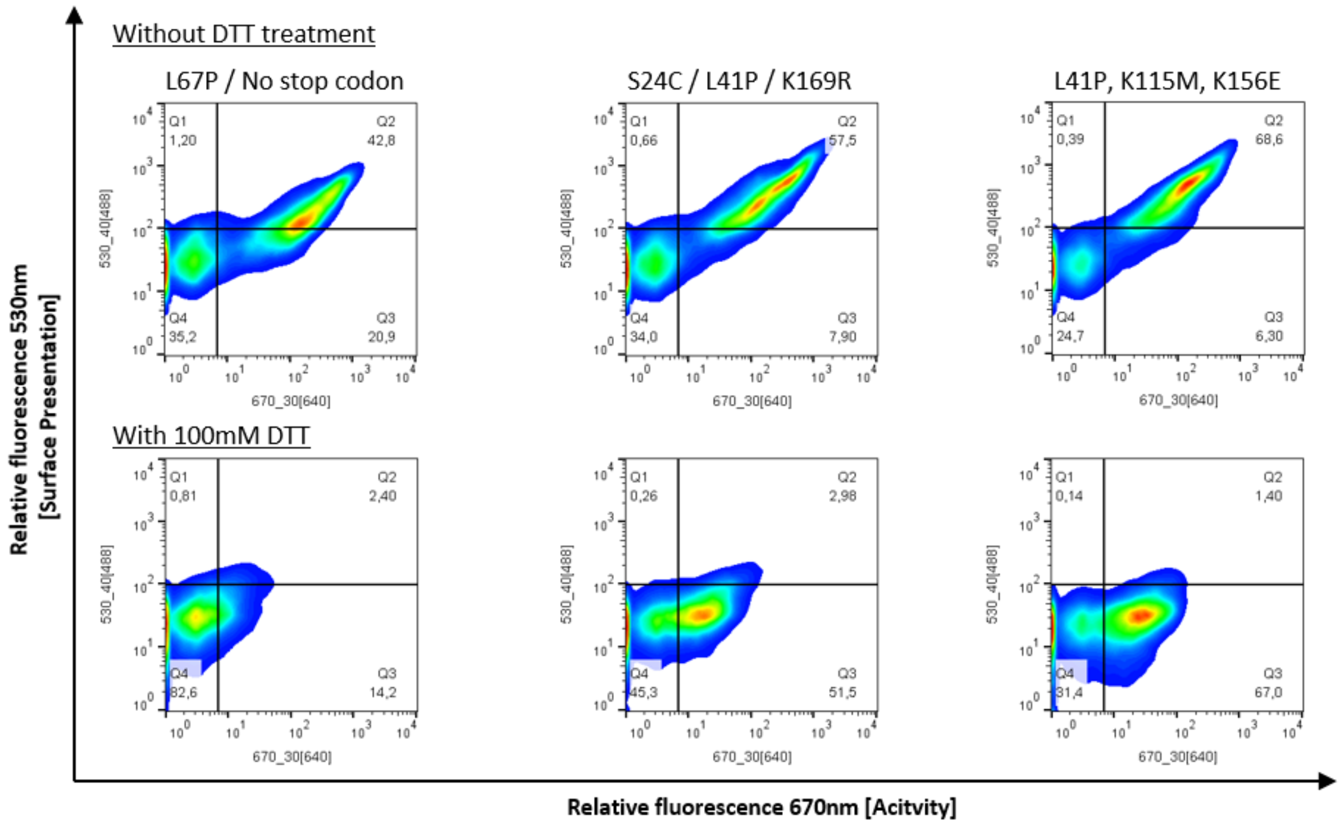

Supplementary Figure 5: Proximity-dependent biotinylation test for the potentially improved microID variants.
The indicated clones were analyzed for surface presentation (y-axis) and biotinylation activity (x-axis). Upper row: analysis after the cell surface biotinylation assay. Lower row: analysis after the cell surface biotinylation and release of the enzymes by reducing the disulfide bonds between Aga1p and Aga2p with DTT.

A

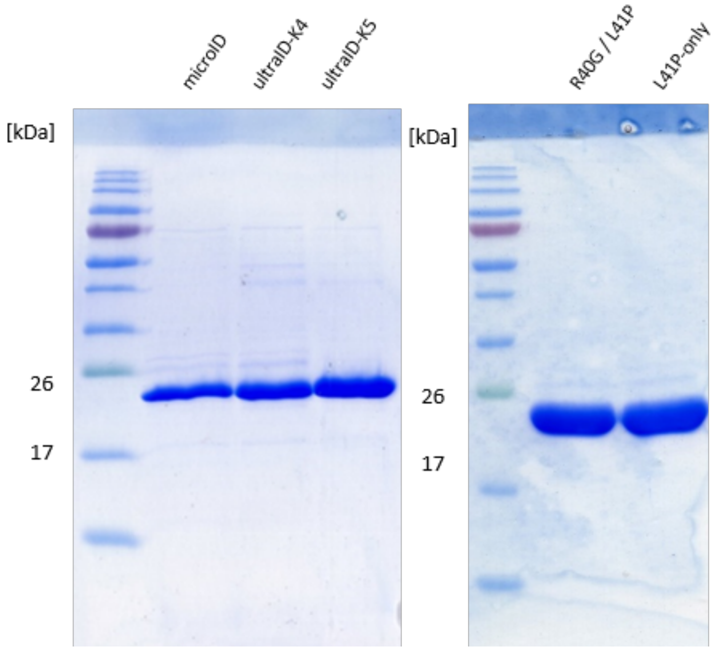

B

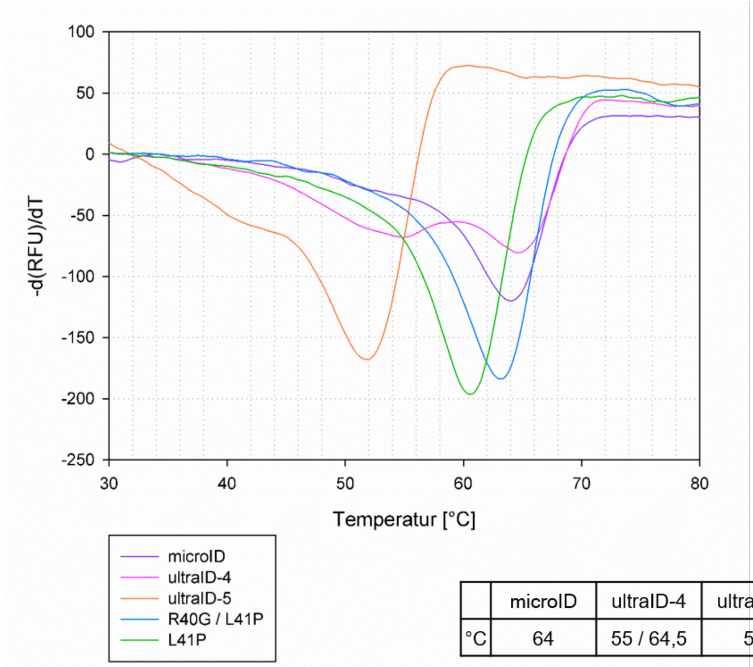

	microID	ultraID-4	ultraID-5	R40G / L41P	L41P
°C	64	55 / 64,5	52	63	60,5

Supplementary Figure 6: Recombinant microID variants and their measured thermostability.
(A) Picture of a Coomassie blue-stained acrylamide gel showing the indicated IMAC affinity purified proteins. (B) Thermostability profile of the purified proteins from (A) determined with a SYPRO orange-based thermal shift assay.

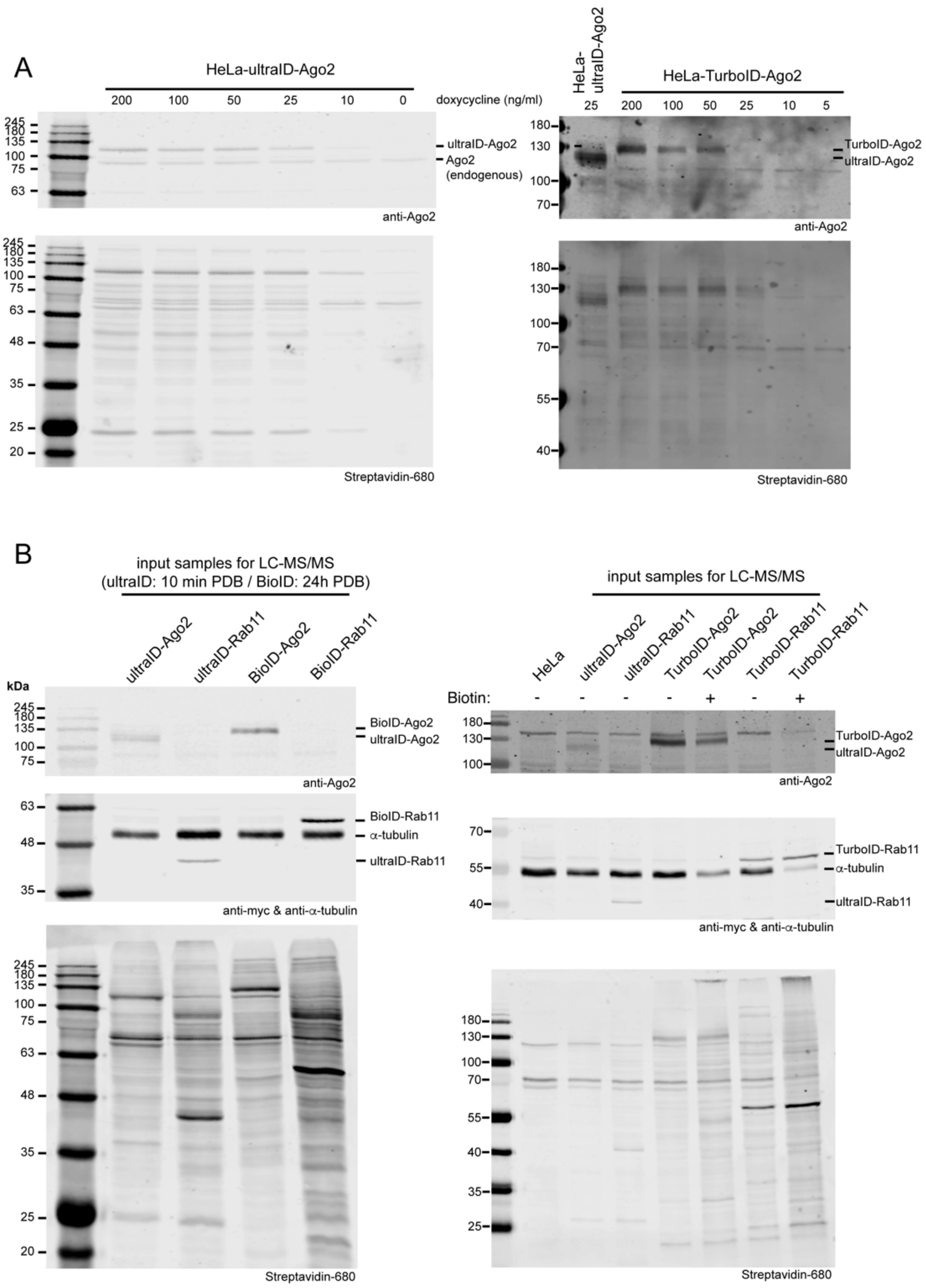

Supplementary Figure 7: Efficient biotinylation with ultra-Ago2 at physiological expression levels.

(A) Blots of lysates of HeLa cells stably expressing Myc-tagged ultraID-Ago2 (left) or TurboID-Ago2 (right) after induction with the indicated concentrations of doxycycline. PDB was induced with biotin for 10 min. (B) Left: Blots of lysates of HeLa cells stably expressing the indicated fusion proteins and used as a starting material for the LC-MS/MS analysis. Biotinylation was analyzed using IRDye680-labeled streptavidin and expression levels of the fusion proteins with antibodies against the Myc tag or Ago2 as indicated. Detection of α-tubulin serves as a loading control on the middle panel. Upper and lower panels belong to the same membrane analyzed with two fluorophores, the endogenous biotinylated protein running at 70 kDa or between the 63 and 75 kDa markers serves as an internal loading control.

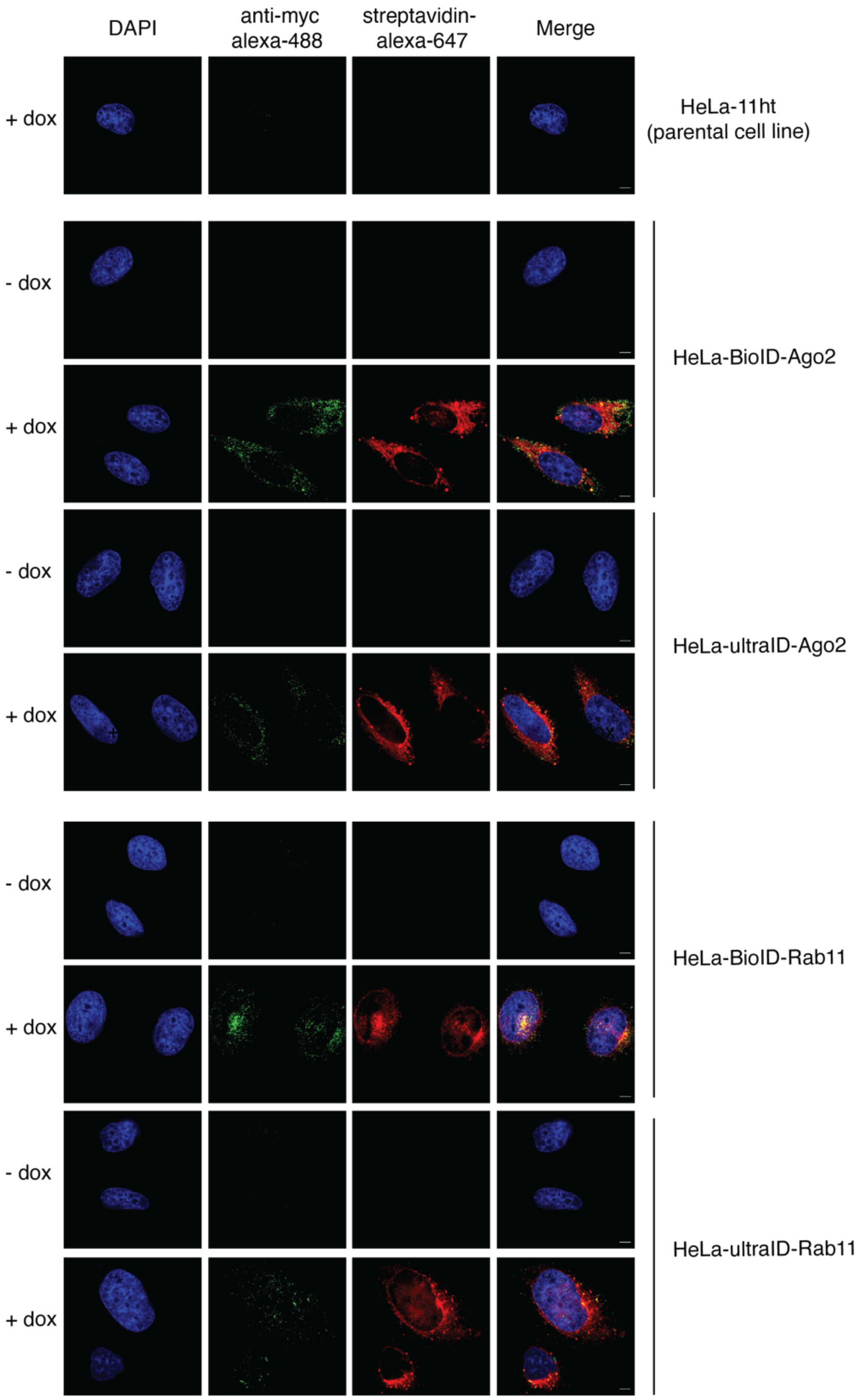

Supplementary Figure 8: Localization of the ultraID-Ago2/Rab11 and BioID-Ago2/Rab11 fusion proteins.
Immunofluorescence of the indicated expressed fusion proteins by Myc-tag detection as well as the detection of biotinylated proteins by alexa-488-coupled streptavidin. Scale bar, 5 µm. All fusion proteins are expressed in the cytosol and show their expected staining pattern.

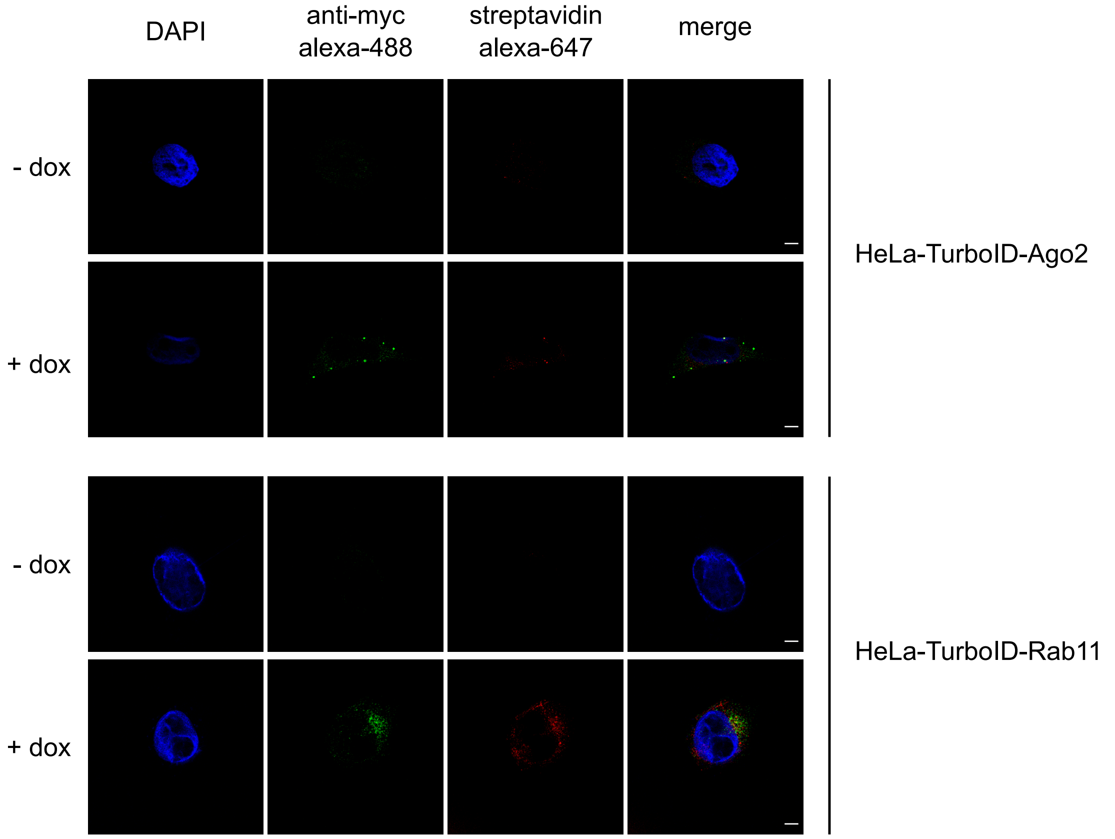

Supplementary Figure 9: Localization of the TurboID-Ago2/Rab11 fusion proteins.
Immunofluorescence of the indicated expressed fusion proteins by Myc-tag detection as well as the detection of biotinylated proteins by alexa-488-coupled streptavidin. Scale bar, 5 µm. All fusion proteins are expressed in the cytosol and show their expected staining pattern.

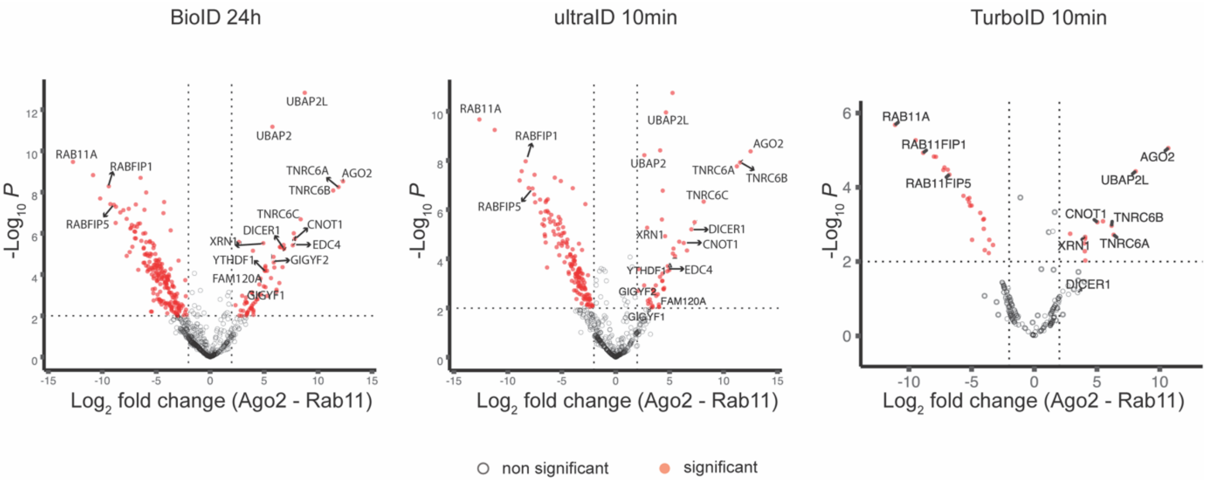

Supplementary Figure 10: cytosolic and membrane proteins proximal to γ1-COP and γ2-COP.

Volcano plots of the proteins identified by LC-MS/MS for the BioID, ultraID and TurboID fusion proteins after PDB as indicated. Significant hits (p-value < 0.01 and $\log_2$ fold change > 2, n = 4) are indicated in red. Selected hits are labeled**.**

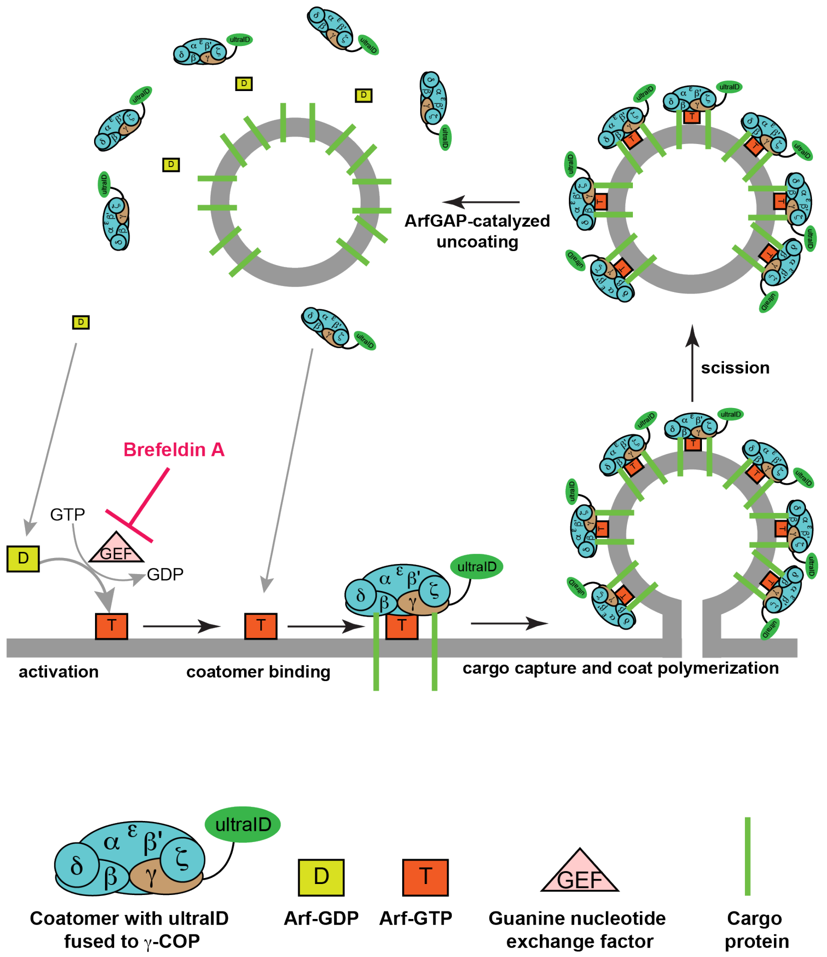

Supplementary Figure 11: Cycle of coatomer recruitment to and release from Golgi membrane.

Cytosolic coatomer is recruited to Golgi membranes by the small GTPase Arf after its activation through GDP to GTP exchange mediated by a specific GEF. Once at the Golgi membrane, the coatomer/Arf complex binds to transmembrane cargo proteins and polymerizes, thereby deforming the membrane until a vesicle pinches off. Soon after membrane scission, GTP-hydrolysis by Arf1 is stimulated by ArfGAPs, leading to Arf-GDP and coatomer release to the cytosol. The drug brefeldin A is a specific inhibitor of the Arf-GEF, thereby blocking the recruitment of coatomer to membranes.

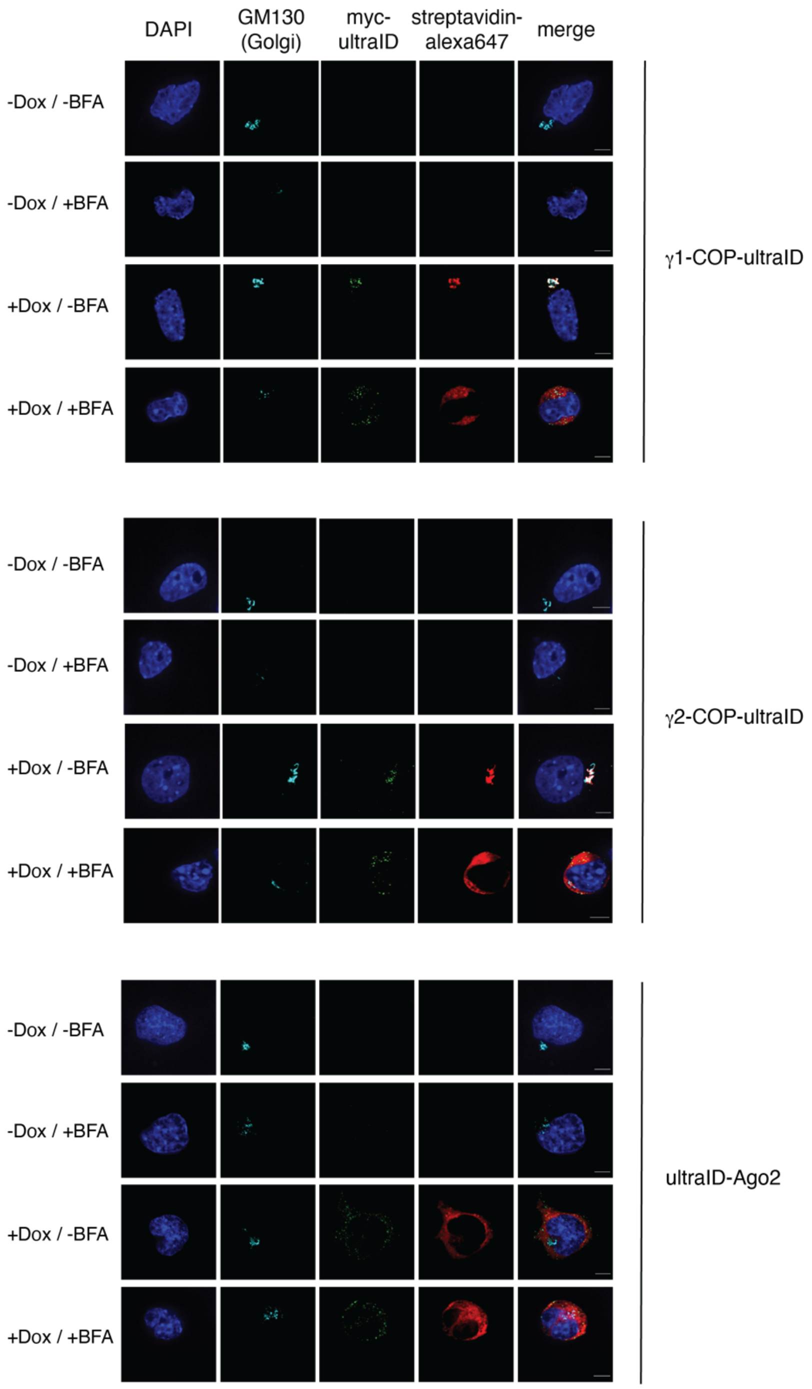

Supplementary Figure 12: Localization of the γ1-COP-ultraID, γ2-COP-ultraID and ultraID-Ago2 fusion proteins.
Immunofluorescence of the indicated expressed fusion proteins by Myc-tag detection and of the Golgi marker GM130. Biotinylated proteins after PDB were detected with alexa-647-coupled streptavidin. Scale bar, 5 µm. The non-induced cell lines (-Dox) served as a staining specificity control. When indicated the cells had been treated with brefeldin A (+BFA).

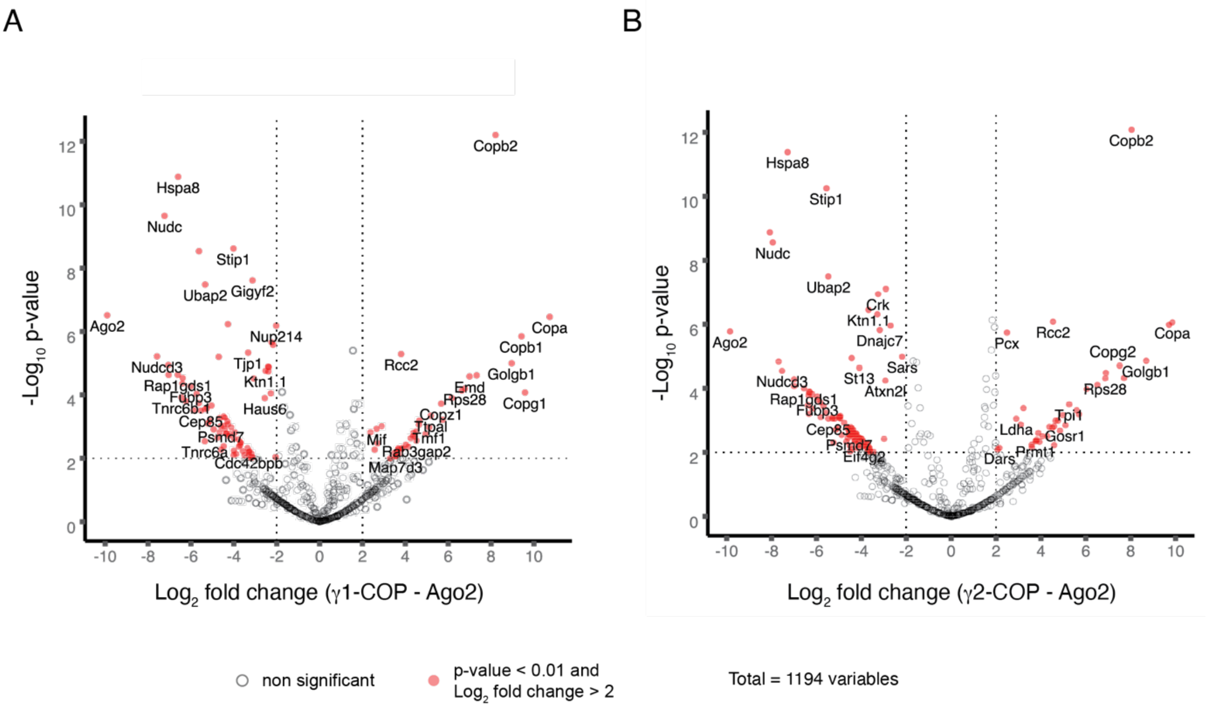

Supplementary Figure 13: cytosolic and membrane proteins proximal to γ1-COP and γ2-COP.

(A) Volcano plots of the proteins identified by LC-MS/MS for the γ1-COP-ultraID protein after PDB under mock conditions. The ultraID-Ago2 dataset served as a negative control. Significant hits (adjusted p-value < 0.01 and $\log_2$ fold change > 2) are indicated in red. Selected hits are labeled. (B) same as (A) for the γ2-COP-ultraID protein

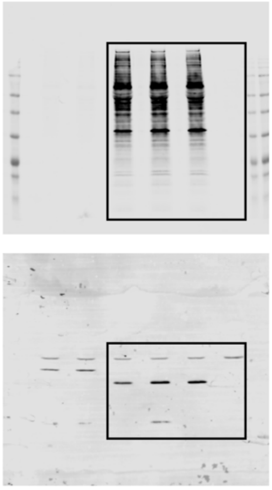

For Fig. 1

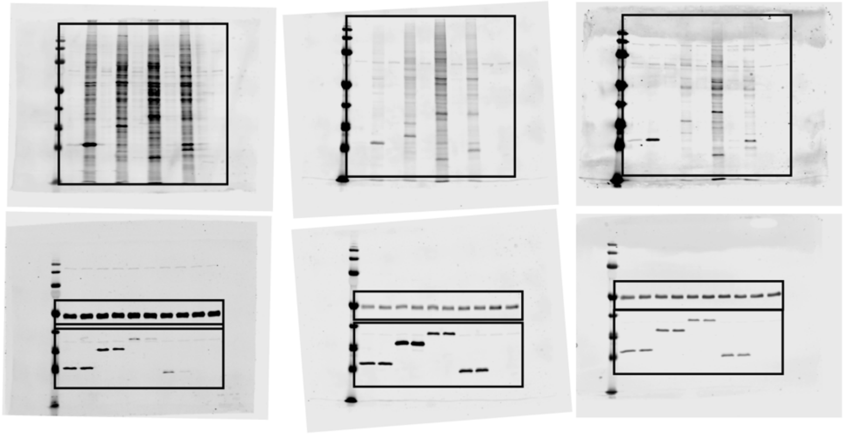

For Fig. 2

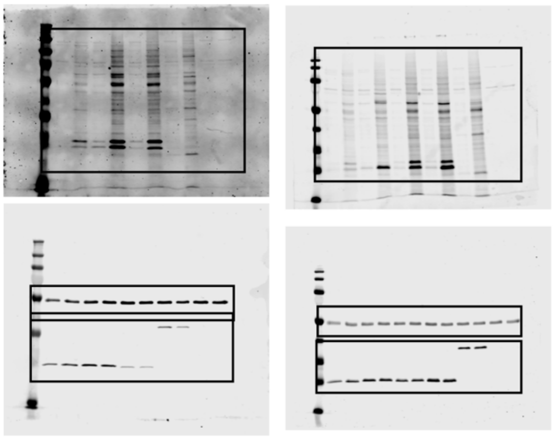

For Fig. 4

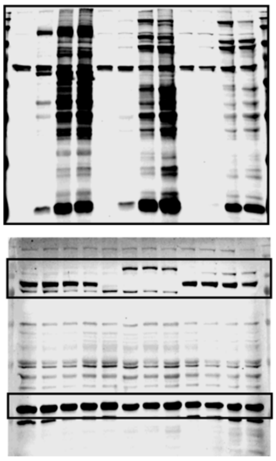

For Fig. 8

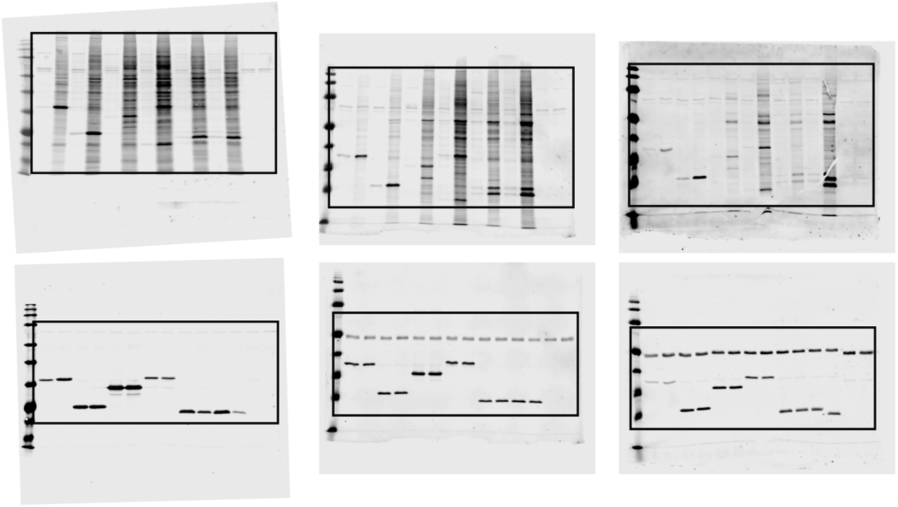

For Fig. 5

Supplementary Figure 14: Full blot scans for Figures 1, 2, 4, 8 and 5

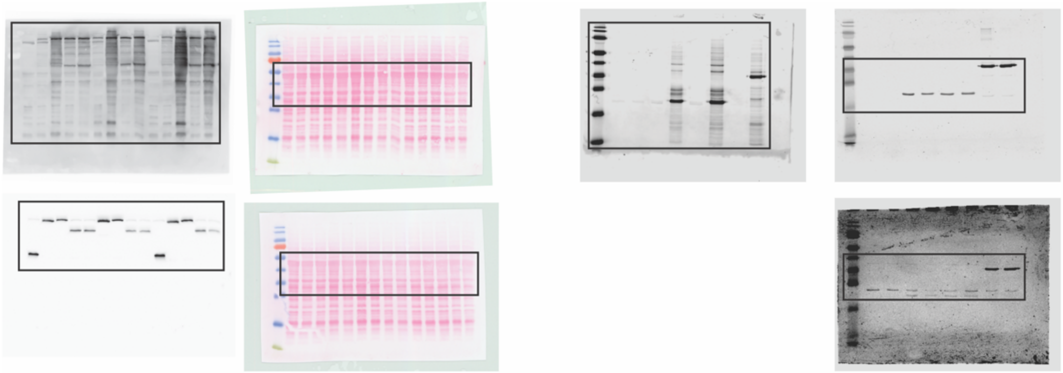

For Fig. 6

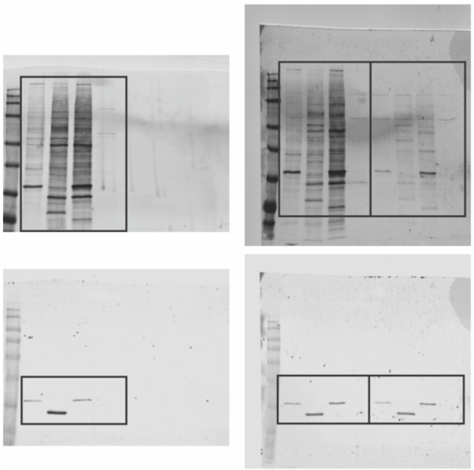

For Supp. Fig. 1

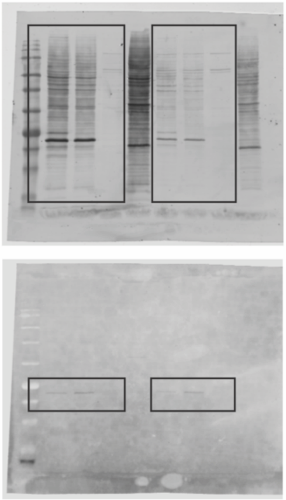

For Supp. Fig. 2

Supplementary Figure 15: Full blot scans for Figure 6 and supplementary figures 1 and 2

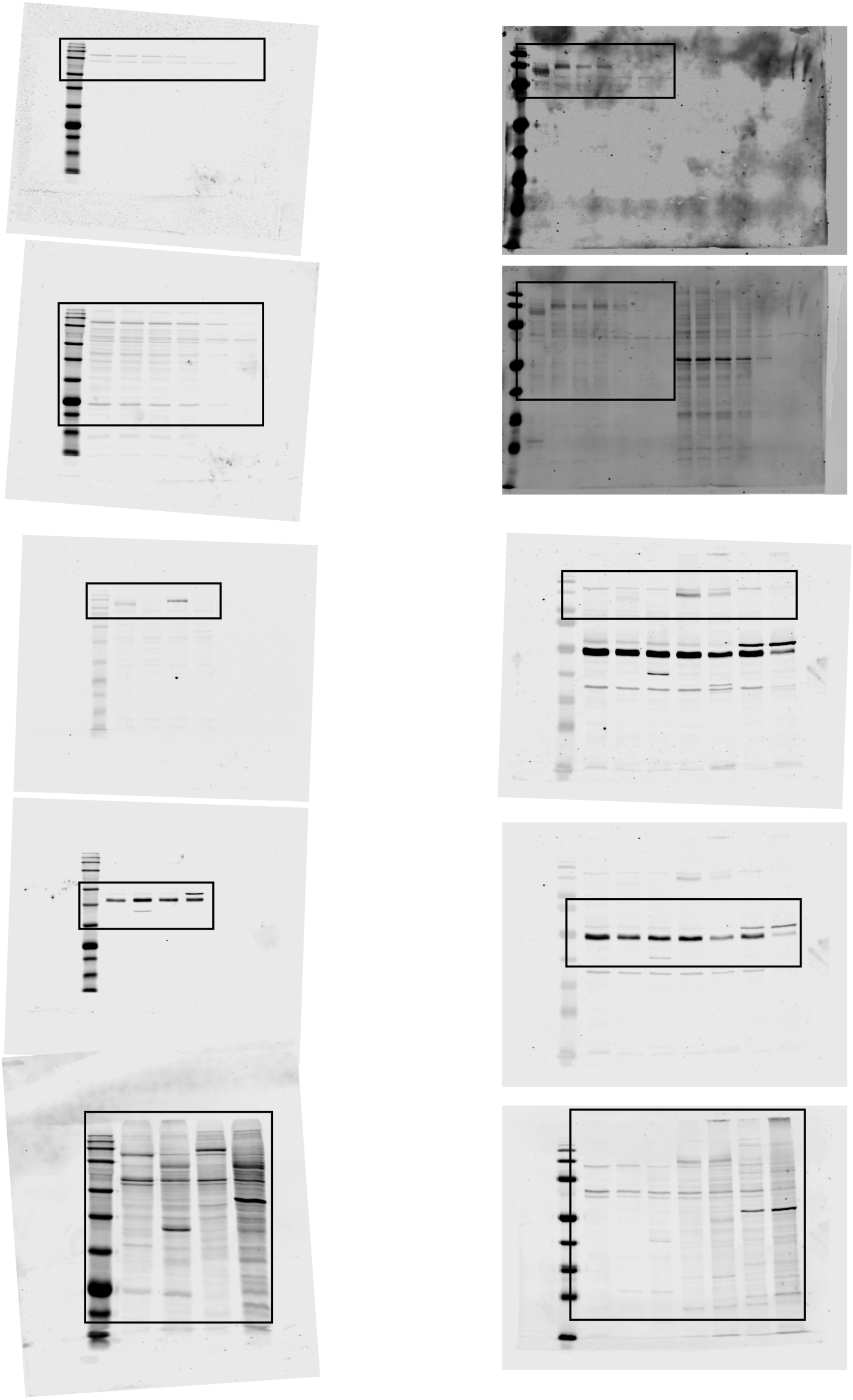

For Supp. Fig. 7

Supplementary Figure 16: Full blot scans for Supplementary Figure 7

Supplementary Table 1: Plasmids generated in this study

Plasmid	Description
pSF3-NBioID2-FKBP/CBioID2-FRB	Co-expression of NBioID2-FKBP and CBioID2-FRB in HeLa-11ht cells
pSF3- NBioID2-FKBP	Expression of NBioID2-FKBP in HeLa-11ht cells
pSF3-BioID	Expression of BioID in HeLa-11ht cells
pSF3-BioID2	Expression of BioID2 in HeLa-11ht cells
pSF3-BASU	Expression of BASU in HeLa-11ht cells
pSF3-TurboID	Expression of TurboID in HeLa-11ht cells
pSF3-microID	Expression of microID in HeLa-11ht cells
pSF3-microID-L41P only	Expression of microID-L41P in HeLa-11ht cells
pSF3-ultraID-4	Expression of ultraID-4 in HeLa-11ht cells
pSF3-ultraID-5	Expression of ultraID-5 in HeLa-11ht cells
pSF3-ultraID	Expression of ultraID in HeLa-11ht cells
pCT-clone 1	Yeast display expression plasmid for clone 1 of the directed evolution selection
pCT-clone 4 (ultraID-4)	Yeast display expression plasmid for clone 4 of the directed evolution selection
pCT-clone 5 (ultraID-5)	Yeast display expression plasmid for clone 5 of the directed evolution selection
pET-22b-microID	Expression of microID in *E. coli*
pET-22b-microID-L41P only	Expression of microID-L41P in *E. coli*
pET-22b-ultraID-4	Expression of ultraID-4 in *E. coli*
pET-22b-ultraID-5	Expression of ultraID-5 in *E. coli*
pET-22b-ultraID	Expression of ultraID in *E. coli*
pET15b-CNOT9	Expression of CNOT9 in *E. coli*
pET15b-microID	Expression of microID in *E. coli*
pET15b-ultraID	Expression of ultraID in *E. coli*
indPB4-γ1-COP-ultraID	PiggyBac-based plasmid for making doxycycline-inducible stable P19 cells for the expression of γ1-COP-ultraID
indPB4-γ2-COP-ultraID	PiggyBac-based plasmid for making doxycycline-inducible stable P19 cells for the expression of γ2-COP-ultraID
indPB4-ultraID-Ago2	PiggyBac-based plasmid for making doxycycline-inducible P19 cells for the expression of ultraID-Ago2
pME2787	*MET25Prom*, *CYC1Term*, *URA3,* 2 µm (reference: Mumberg et al., 1994)
pME4478	*MET25Prom*, *CYC1Term*, *URA3,* 2 µm, *ASC1-birA** (reference: Opitz *et al*. 2017)
pME5086	*MET25Prom*, *CYC1Term*, *URA3,* 2 µm, *ASC1-µID*
pME5087	*MET25Prom*, *CYC1Term*, *URA3,* 2 µm, *ASC1-ultraID*
pME5479	*MET25Prom*, *CYC1Term*, *URA3,* 2 µm, *ASC1-TurboID* (Template for TurboID: TurboID-V5_pRS415 (Addgene plasmid #107167)
pSF3-ultraID-Ago2	For making doxycycline-inducible stable HeLa 11ht cells for the expression of ultraID-Ago2
pSF3-ultraID-Rab11	For making doxycycline-inducible stable HeLa 11ht cells for the expression of ultraID-Rab11

Supplementary Table 2: *S. cerevisiae* strains used in this work

Strain	Genotype	Reference
RH2817	*MATα, ura3-52, trp1::hisG*	Valerius *et al*., 2007
RH3263	*MATα, ura3-52, trp1::hisG, leu2::hisG, Δasc1::LEU2*	Valerius *et al*., 2007

Supplementary Table 3: Primers used in this work

Primer	Sequence 5'-> 3'
microID library gap up	GTGGTGGTGGTTCTGGTGGTGGTGGTTCTGaacaaaaactcatctcagaagaggatctc
microID library gap lo	tcTACACTGTTGTTATCAGATCTCGAgCTATTAatggtgatggtgatgatg
ultraID NcoI up	ataataccatgggcGAACAAAAACTCATCTCAGAAGAGGATCTC
ultraID EcoRI lo	tattatgaattcTTAATGGTGATGGTGATGATGCTTCTC
microID SDM R40G/L41P	GGGCAGAGGCGGCCCGGGCAGAAAGTGGCTGAGCCAGG
microID SDM G40R/L41P	GGGCAGAGGCCGCCCGGGCAGAAAGTGGCTGAGCCAGG
microID SDM lo	TTGGTCTGTCTGTCGGCCACC

Supplementary Table 4: Antibodies used in this study

Antibody	Host	Source	Dilution
Anti-myc (9E10)	mouse	DSHB (sc-40X)	WB, 1:1‘000
Anti-FLAG (M2)	mouse	Sigma (F1804)	WB, 1:500
Anti-CNOT9	rabbit	Proteintech (22503-1 AP)	WB, 1 :3’000
Anti-α-tubulin (B-5-1-2)	mouse	Sigma (T5168)	WB, 1:10‘000
Anti-Ago2 (11A9)	rat	Sigma (MABE253)	WB, 1:5‘000
anti-γ1-COP (anti-γ1-app)	rabbit	Wieland lab (Heidelberg)	WB, 1:800
anti-γ2-COP (anti-γ2-app)	rabbit	Wieland lab (Heidelberg)	WB, 1:800
anti-Asc1p	rabbit	Valerius lab	WB, 1:1‘000
anti-myc-Tag (71D10)	rabbit	Cell signalling technology (2278S)	IF, 1:400
Anti-GM130 (35/GM130)	mouse	BD Transduction Laboratories (610822)	IF, 1:500
anti-mouse IgG IRDye 800CW	goat	LI-COR (C40826-01)	WB, 1:15‘000
anti-rat IgG DyLight800	goat	Thermo Scientific (SA5-10024)	WB, 1:15‘000
anti-mouse IgG AlexaFluor680	goat	Thermo Scientific (A-21057)	WB, 1:10‘000
anti-rabbit IgG IRDye 800CW	goat	LI-COR (926-32211)	WB, 1:15‘000
anti-rabbit IgG IRDye 680CW	goat	LI-COR (926-68071)	WB, 1:15‘000
anti-mouse Alexa 647	donkey	Invitrogen (A28175)	IF, 1:1‘000
anti-mouse Alexa 546	goat	Invitrogen (A11030)	IF, 1:1‘000
anti-rabbit Alexa 488	goat	Invitrogen (A11008)	IF, 1:1‘000
streptavidin-DyLight680	-	Invitrogen (21848)	WB, 1:15‘000
streptavidin-AlexaFluor647	-	Jackson ImmunoResearch (016-600-084)	IF, 1:1‘000

Supplementary Note 1

Typical MS Data analysis for PDB-MS experiments with R and proDA

Firstly, the necessary packages are loaded in R:

```
library(knitr)
library(DEP)
library(dplyr)
library(proDA)
library(rmarkdown)
```

Next, set the working directory.

```
setwd("~/Desktop/BZH Béthune/data/mass spec data/10t”)
```

Analysis pipeline

1) Load MaxQuant data - Save the proteinGroups.txt file from MaxQuant in a folder of your choice (This folder will be your “working directory”) - Load the MaxQuant data as follow:

```
full_data10t<-read.delim("proteinGroups.txt", stringsAsFactors = FALSE)
```

2) Filter the data in full_data10t for contaminants, reverse, and only identified by modification

```
full_data10t <- filter(full_data10t, Reverse != "+", Potential.contaminant
!= "+", Only.identified.by.site != "+")
```

Only works when dplyr loaded! Filter() allows you to select a subset of rows, here you say: filter the raws in the file full_data for which the value for Reverse, potential contaminant, and only identified by site is not equal to (!=) to “+”, save in full_data10t

3) Check if you have rows that have the same gene names (this is usually the case)

```
full_data10t$Gene.names %>% duplicated() %>% any()
## [1] TRUE
```

3) Make the gene names uniques (e.g. if you have Act twice, the first one will be called Act.1, the second Act.2)

```
data_unique10t <- make_unique(full_data10t, "Gene.names", "Protein.IDs",
delim = ";")
```

4) Create an experiment design file In this step we will create a file that describes the experiment design (what samples, which conditions, how many replicates), - Create a list that contains the column names with from the LFQ columns and check the correct assignment:

```
replicate_names10t<-c(colnames(data_unique10t)[71:78])
print(replicate_names10t)
##  [1]
"LFQ.intensity.10tAgo2_1"  "LFQ.intensity.10tAgo2_2"  "LFQ.intensity.10tAgo
2_3"
##  [4] "LFQ.intensity.10tAgo2_4"  "LFQ.intensity.10tRab11_1"
"LFQ.intensity.10tRab11_2"
##  [7] "LFQ.intensity.10tRab11_3" "LFQ.intensity.10tRab11_4"
```

- Create the experimental file as the following example:

```
exp_design10t <- data.frame(
label = replicate_names10t,
```

```
condition = c("10tAgo2", "10tAgo2", "10tAgo2","10tAgo2", "10tRab11",
"10tRab11", "10tRab11", "10tRab11"), replicate=rep(1:4, times=2),
stringAsFactors = FALSE)
```

Then check your experiment design file:

```
exp_design10t
##                     label  condition         replicate
      stringAsFactors
## 1   LFQ.intensity.10tAgo2_1     10tAgo2         1           FALSE
## 2   LFQ.intensity.10tAgo2_2     10tAgo2         2           FALSE
## 3   LFQ.intensity.10tAgo2_3     10tAgo2         3           FALSE
## 4   LFQ.intensity.10tAgo2_4     10tAgo2         4           FALSE
## 5  LFQ.intensity.10tRab11_1    10tRab11         1           FALSE
## 6  LFQ.intensity.10tRab11_2    10tRab11         2           FALSE
## 7  LFQ.intensity.10tRab11_3    10tRab11         3           FALSE
## 8  LFQ.intensity.10tRab11_4    10tRab11         4           FALSE
```

5) Import your prepared MaxQuant data into a "summarized experiment" file

```
 se10t <- import_MaxQuant(data_unique10t, exp_design10t)
```

6) Fit the probability drop out model to your data (this takes several minutes!)

```
fit10t <- proDA(se10t, design = ~ condition - 1, max_iter = 200)
```

and check that the fit converged :

```
fit10t
##  Parameters of the probabilistic dropout model
##
## The dataset contains 8 samples and 393 proteins
## 77.4% of the values are missing
##
## Experimental design: y~condition - 1
## The model has successfully converged.
##
## The inferred parameters are:
## location_prior_mean:     19.4
## location_prior_scale:    14.3
## location_prior_df:       3
## variance_prior_scale:    0.434
## variance_prior_df:       0.955
## dropout_curve_position:  20.9, 20.7, 23.4, 21.2, ...
## dropout_curve_scale:     -0.564, -0.843, -1.58, -0.731, ...
```

7) Apply the fit to test differential expression

Example for Ago2 vs. Rab11:

```
10tAgo2_test <- proDA::test_diff(fit10t, contrast = conditionX10tAgo2 -
conditionX10tRab11)
```

To save the file on your computer:

```
write.csv(10tAgo2_test, file = "Ago2 vs Rab11 TurboID.csv")
```

This will create the Ago2 vs Rab11 ultraID.csv file in your working directory.
