# Supplementary material for: Engineering of ultraID, a compact and hyperactive enzyme for proximity-dependent biotinylation in living cells
Source: Commun Biol. 2022 Jul 4;5:657. doi: 10.1038/s42003-022-03604-5 (PMC9253107; doi:10.1038/s42003-022-03604-5)
Supplement: Supplementary file 3 — Description of Additional Supplementary Files [file 42003_2022_3604_MOESM3_ESM.pdf]

Description of Additional Supplementary Files

File name: Supplementary Data 1
Description: Mass spectrometry Data for the PDB experiments with HeLaBioID (24h labeling) and ultraID (10 min labeling) cell lines.

File name: Supplementary Data 2
Description: Mass spectrometry Data for the PDB experiments with HeLaultraID (no labeling) and TurboID (no and 10 min labeling) cell lines.

File name: Supplementary Data 3
Description: Mass spectrometry Data for the PDB experiments with P19- γ1- COP-ultraID and P19-γ2-COP-ultraID cell lines.

File name: Supplementary Data 4
Description: Raw data for the ELISA activity assay.

File name: Supplementary Data 5
Description: Raw data for the melting temperature assay.
