# Supplementary material for: Engineering of ultraID, a compact and hyperactive enzyme for proximity-dependent biotinylation in living cells
Source: Commun Biol. 2022 Jul 4;5:657. doi: 10.1038/s42003-022-03604-5 (PMC9253107; doi:10.1038/s42003-022-03604-5)
Supplement: Supplementary file 9 — Reporting Summary [file 42003_2022_3604_MOESM9_ESM.pdf]

nature research

Corresponding author(s): Julien Béthune

Last updated by author(s): May 5, 2022

Reporting Summary

Nature Research wishes to improve the reproducibility of the work that we publish. This form provides structure for consistency and transparency in reporting. For further information on Nature Research policies, see our Editorial Policies and the Editorial Policy Checklist.

Statistics

For all statistical analyses, confirm that the following items are present in the figure legend, table legend, main text, or Methods section.

n/a	Confirmed	
☐	☒	The exact sample size (*n*) for each experimental group/condition, given as a discrete number and unit of measurement
☐	☒	A statement on whether measurements were taken from distinct samples or whether the same sample was measured repeatedly
☐	☒	The statistical test(s) used AND whether they are one- or two-sided<br>*Only common tests should be described solely by name; describe more complex techniques in the Methods section.*
☒	☐	A description of all covariates tested
☐	☒	A description of any assumptions or corrections, such as tests of normality and adjustment for multiple comparisons
☐	☒	A full description of the statistical parameters including central tendency (e.g. means) or other basic estimates (e.g. regression coefficient) AND variation (e.g. standard deviation) or associated estimates of uncertainty (e.g. confidence intervals)
☐	☒	For null hypothesis testing, the test statistic (e.g. F, t, r) with confidence intervals, effect sizes, degrees of freedom and P value noted<br>*Give P values as exact values whenever suitable.*
☒	☐	For Bayesian analysis, information on the choice of priors and Markov chain Monte Carlo settings
☒	☐	For hierarchical and complex designs, identification of the appropriate level for tests and full reporting of outcomes
☒	☐	Estimates of effect sizes (e.g. Cohen's *d*, Pearson's *r*), indicating how they were calculated

Our web collection on statistics for biologists contains articles on many of the points above.

Software and code

Policy information about availability of computer code

Data collection	MS data collection: Q Exactive HF mass spectrometer and XCalibur 4.0 software (both Thermo Fisher Scientific)
Data analysis	MS data analysis: MaxQuant version 1.5.5.1 software (Cox, J. & Mann, M, doi: 10.1038/nbt.1511, see maxquant.org)<br>MS data analysis: proDA (R-package from Ahlmann-Eltze C & Anders S, doi: 10.1101/661496, see: https://github.com/const-ae/proDA)

For manuscripts utilizing custom algorithms or software that are central to the research but not yet described in published literature, software must be made available to editors and reviewers. We strongly encourage code deposition in a community repository (e.g. GitHub). See the Nature Research guidelines for submitting code & software for further information.

Data

Policy information about availability of data

All manuscripts must include a data availability statement. This statement should provide the following information, where applicable:

- Accession codes, unique identifiers, or web links for publicly available datasets
- A list of figures that have associated raw data
- A description of any restrictions on data availability

The MS proteomics data have been deposited to the ProteomeXchange Consortium (http://proteomecentral.proteomexchange.org) via the PRIDE partner repository with the data set identifier PXD026719, PXD026715 and PXD032979

Field-specific reporting

Please select the one below that is the best fit for your research. If you are not sure, read the appropriate sections before making your selection.

☒ Life sciences ☐ Behavioural & social sciences ☐ Ecological, evolutionary & environmental sciences

For a reference copy of the document with all sections, see nature.com/documents/nr-reporting-summary-flat.pdf

Life sciences study design

All studies must disclose on these points even when the disclosure is negative.

Sample size	This is a cell culture-based study in which millions of cells from a homogeneous population are tested in parallel. Appropriate power to discovery of true positive events was gained from three to four biological replicates.
Data exclusions	no data were excluded from the analyses
Replication	Western blot analyses were replicated at least three times, proximity-dependent labeling/MS experiments were performed on at least three biological replicates per samples. All attempts at replication were successful.
Randomization	Non relevant: millions of cells from homogeneous populations were tested in parallel
Blinding	The samples for MS analysis were given reference numbers that did not allow for identification

Reporting for specific materials, systems and methods

We require information from authors about some types of materials, experimental systems and methods used in many studies. Here, indicate whether each material, system or method listed is relevant to your study. If you are not sure if a list item applies to your research, read the appropriate section before selecting a response.

Materials & experimental systems

n/a	Involved in the study	
☐	☒	Antibodies
☐	☒	Eukaryotic cell lines
☒	☐	Palaeontology and archaeology
☒	☐	Animals and other organisms
☒	☐	Human research participants
☒	☐	Clinical data
☒	☐	Dual use research of concern

Methods

n/a	Involved in the study	
☒	☐	ChIP-seq
☐	☒	Flow cytometry
☒	☐	MRI-based neuroimaging

Antibodies

Antibodies used	Primaries:<br>Anti-myc (9E10, Santa Cruz # sc-40x), Anti-FLAG (M2, Sigma # F1804), Anti-CNOT9 (Proteintech #22503-1 AP), Anti-alpha-tubulin (B-5-1-2, Sigma # T5168), anti-Ago2 (11A9, Sigma #MABE253), anti-Myc-tag (71D10, CST #2278S), anti-GM130 (35/GM130, BD transduction laboratories #610822), anti-gamma1-COP and anti-gamma2-COP (both from Felix Wieland's lab, BZH Heidelberg, Germany).<br>Secondaries:<br>anti-mouse IgG IRDye800CW (Li-Cor #C40826), anti-rat IgG DyLight800 (Thermo Scientific #SA5-10024), anti-mouse IgG-alexa680 (Thermo Scientific #SA5-21057), anti-rabbit IgG IRDye800CW (Li-Cor #926-32211), anti-rabbit IgG IRDye680CW (Li-Cor #926-68071), anti-mouse IgG-Alexa 647 (Invitrogen #A28175), anti-mouse IgG-Alexa 546 (Invitrogen #A11030), anti-rabbit IgG-Alexa 488 (Invitrogen #A11008)
Validation	All commercial antibodies were validated by the manufacturers. The anti-gamma1-COP and anti-gamma2-COP antibodies were validated by us using KO P19 cell lines (Jain Goyal et al., LSA 2020)

Eukaryotic cell lines

Policy information about cell lines

Cell line source(s)	HeLa EM2-11ht (aka HeLa-11ht) obtained from its lab of origin (Kai Schönig, ZI Mannheim, Germany)<br>P19 cells (purchased from Sigma Aldrich)
Authentication	The cell lines were not authenticated but MS analysis confirmed correct species

Mycoplasma contamination	All cell lines were regularly tested negative for mycoplasma contamination
Commonly misidentified lines (See ICLAC register)	none

Flow Cytometry

Plots

Confirm that:

☒ The axis labels state the marker and fluorochrome used (e.g. CD4-FITC).

☒ The axis scales are clearly visible. Include numbers along axes only for bottom left plot of group (a 'group' is an analysis of identical markers).

☒ All plots are contour plots with outliers or pseudocolor plots.

☒ A numerical value for number of cells or percentage (with statistics) is provided.

Methodology

Sample preparation	1×107 - 1×108 yeasts were washed twice and resuspended in 1 mL PBS. Biotin and ATP were added to a final concentration of 50 µM and 2.5 mM, respectively. Cells were incubated at 30°C and 900 rpm to prevent settling. Incubation time was decreased for each screening round. To prepare yeast cells for FACS screening, biotinylated cells were washed twice with PBS, resuspended in 100 µL PBS containing 0.1 µg anti-penta·His antibody (Qiagen) and 1 µg streptavidin allophycocyanin (Invitrogen) and incubated on ice for 20 min. After two washing steps, cells were stained as described before using 0.2 µg anti-mouse FITC antibody (Sigma-Aldrich). Finally, cells were washed again twice and were suspended in 1.5 mL of PBS.
Instrument	BD Influx system
Software	BD FACS software 1.0.0.650
Cell population abundance	*Describe the abundance of the relevant cell populations within post-sort fractions, providing details on the purity of the samples and how it was determined.*
Gating strategy	*Describe the gating strategy used for all relevant experiments, specifying the preliminary FSC/SSC gates of the starting cell population, indicating where boundaries between "positive" and "negative" staining cell populations are defined.*

☒ Tick this box to confirm that a figure exemplifying the gating strategy is provided in the Supplementary Information.
